# Supplementary material for: iTRAQ-Based Proteomics Analysis of Response to Solanum tuberosum Leaves Treated with the Plant Phytotoxin Thaxtomin A
Source: Int J Mol Sci. 2021 Nov 7;22(21):12036. doi: 10.3390/ijms222112036 (PMC8585116; doi:10.3390/ijms222112036)
Supplement: Supplementary file 1 [file ijms-22-12036-s001.zip › ijms-1405429-supplementary/Figure S1 Protein interaction network.pdf]

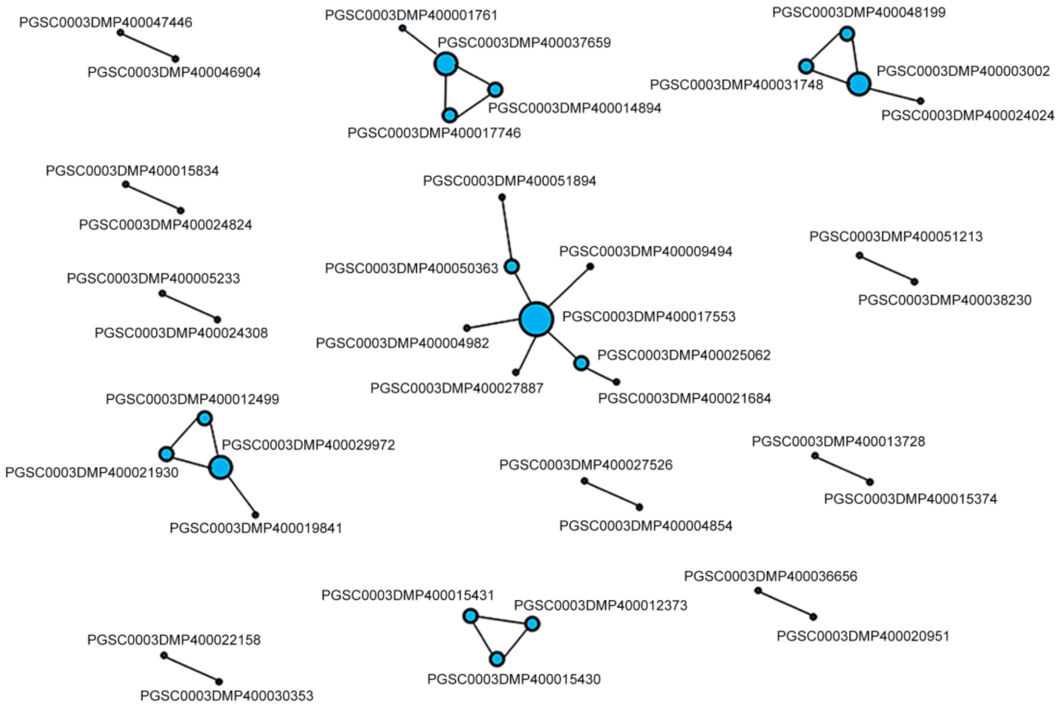

Figure S1 Protein interaction network. Each node represents a protein, and the line between nodes represents the interaction between the two proteins. The size of the node represents the number of the interacting proteins.
