# Supplementary material for: iTRAQ-Based Proteomics Analysis of Response to Solanum tuberosum Leaves Treated with the Plant Phytotoxin Thaxtomin A
Source: Int J Mol Sci. 2021 Nov 7;22(21):12036. doi: 10.3390/ijms222112036 (PMC8585116; doi:10.3390/ijms222112036)
Supplement: Supplementary file 1 [file ijms-22-12036-s001.zip › ijms-1405429-supplementary/Table S1. GO enrichment analysis of DEPs.pdf]

Table S1. GO enrichment analysis of DEPs

GO Description	GO Term(Level1)	GO ID	Protein Number
Biological_process	BP	GO:0008150	392
Single-organism process	BP	GO:0044699	204
Single-organism metabolic process	BP	GO:0044710	166
Oxidation-reduction process	BP	GO:0055114	99
Response to stimulus	BP	GO:0050896	65
Response to stress	BP	GO:0006950	53
Single-organism catabolic process	BP	GO:0044712	29
Protein folding	BP	GO:0006457	21
Defense response	BP	GO:0006952	18
Response to oxidative stress	BP	GO:0006979	17
Detoxification	BP	GO:0098754	17
Cellular oxidant detoxification	BP	GO:0098869	17
Cellular detoxification	BP	GO:1990748	17
Protein complex subunit organization	BP	GO:0071822	15
Photosynthesis	BP	GO:0015979	15
Organonitrogen compound catabolic process	BP	GO:1901565	14
Response to biotic stimulus	BP	GO:0009607	13
Protein complex assembly	BP	GO:0006461	13
Cell redox homeostasis	BP	GO:0045454	13
Monocarboxylic acid biosynthetic process	BP	GO:0072330	12
Negative regulation of molecular function	BP	GO:0044092	11
Aromatic amino acid family metabolic process	BP	GO:0009072	11
Reactive oxygen species metabolic process	BP	GO:0072593	10
Negative regulation of catalytic activity	BP	GO:0043086	10
Small molecule catabolic process	BP	GO:0044282	9
Electron transport chain	BP	GO:0022900	9
Cellular protein complex assembly	BP	GO:0043623	9
Photosynthetic electron transport chain	BP	GO:0009767	8

Organic acid catabolic process	BP	GO:0016054	8
Hydrogen peroxide metabolic process	BP	GO:0042743	8
Hydrogen peroxide catabolic process	BP	GO:0042744	8
Carboxylic acid catabolic process	BP	GO:0046395	8
Aromatic compound catabolic process	BP	GO:0019439	8
Regulation of proteolysis	BP	GO:0030162	7
Regulation of peptidase activity	BP	GO:0052547	7
Regulation of endopeptidase activity	BP	GO:0052548	7
Negative regulation of proteolysis	BP	GO:0045861	7
Negative regulation of protein metabolic process	BP	GO:0051248	7
Negative regulation of peptidase activity	BP	GO:0010466	7
Negative regulation of hydrolase activity	BP	GO:0051346	7
Negative regulation of endopeptidase activity	BP	GO:0010951	7
Negative regulation of cellular protein metabolic process	BP	GO:0032269	7
Lipid catabolic process	BP	GO:0016042	7
Carbohydrate derivative catabolic process	BP	GO:1901136	7
Secondary metabolic process	BP	GO:0019748	6
Response to bacterium	BP	GO:0009617	6
Phenylpropanoid metabolic process	BP	GO:0009698	6
Glycerol ether metabolic process	BP	GO:0006662	6
Ether metabolic process	BP	GO:0018904	6
Defense response to bacterium	BP	GO:0042742	6
Cellular amino acid catabolic process	BP	GO:0009063	6
Aromatic amino acid family biosynthetic process	BP	GO:0009073	6
Alpha-amino acid catabolic process	BP	GO:1901606	6
Alcohol metabolic process	BP	GO:0006066	6
Secondary metabolite biosynthetic process	BP	GO:0044550	5
Phenylpropanoid biosynthetic process	BP	GO:0009699	5
L-phenylalanine metabolic process	BP	GO:0006558	5
Erythrose 4-phosphate/phosphoenolpyruvate family amino acid metabolic process	BP	GO:1902221	5

Benzene-containing compound metabolic process	BP	GO:0042537	5
Aromatic amino acid family catabolic process	BP	GO:0009074	5
Response to water deprivation	BP	GO:0009414	4
Response to light intensity	BP	GO:0009642	4
Reductive pentose-phosphate cycle	BP	GO:0019253	4
Photosynthetic electron transport in photosystem II	BP	GO:0009772	4
Photosynthesis, dark reaction	BP	GO:0019685	4
Photorespiration	BP	GO:0009853	4
L-phenylalanine catabolic process	BP	GO:0006559	4
Erythrose 4-phosphate/phosphoenolpyruvate family amino acid catabolic process	BP	GO:1902222	4
Cinnamic acid metabolic process	BP	GO:0009803	4
Cinnamic acid biosynthetic process	BP	GO:0009800	4
Chorismate metabolic process	BP	GO:0046417	4
Eesponse to high light intensity	BP	GO:0009644	3
Eegulation of photosynthesis, light reaction	BP	GO:0042548	3
Eegulation of generation of precursor metabolites and energy	BP	GO:0043467	3
Photosynthesis, light reaction	BP	GO:0019684	3
Oxylipin metabolic process	BP	GO:0031407	3
Oxylipin biosynthetic process	BP	GO:0031408	3
NADH dehydrogenase complex assembly	BP	GO:0010257	3
Jasmonic acid metabolic process	BP	GO:0009694	3
Jasmonic acid biosynthetic process	BP	GO:0009695	3
Cytochrome complex assembly	BP	GO:0017004	3
Chorismate biosynthetic process	BP	GO:0009423	3
Cellular response to radiation	BP	GO:0071478	3
Cellular response to light stimulus	BP	GO:0071482	3
Cellular response to abiotic stimulus	BP	GO:0071214	3
Response to endoplasmic reticulum stress	BP	GO:0034976	2
Regulation of transporter activity	BP	GO:0032409	2
Regulation of transmembrane transporter activity	BP	GO:0022898	2

Regulation of proton transport	BP	GO:0010155	2
Regulation of ion transmembrane transporter activity	BP	GO:0032412	2
Regulation of anion channel activity	BP	GO:0010359	2
Protein K63-linked ubiquitination	BP	GO:0070534	2
Phototropism	BP	GO:0009638	2
Nucleoside catabolic process	BP	GO:0009164	2
NADH dehydrogenase complex (plastoquinone) assembly	BP	GO:0010258	2
Modification of morphology or physiology of other organism	BP	GO:0035821	2
Glycosyl compound catabolic process	BP	GO:1901658	2
Glutamine biosynthetic process	BP	GO:0006542	2
Cytochrome b6f complex assembly	BP	GO:0010190	2
Cellular response to light intensity	BP	GO:0071484	2
Membrane	CC	GO:0016020	74
Chloroplast part	CC	GO:0044434	59
Plastid part	CC	GO:0044435	59
Thylakoid part	CC	GO:0044436	49
Photosynthetic membrane	CC	GO:0034357	37
Thylakoid membrane	CC	GO:0042651	37
Plastid thylakoid membrane	CC	GO:0055035	37
Chloroplast thylakoid membrane	CC	GO:0009535	37
Extracellular region	CC	GO:0005576	20
Thylakoid lumen	CC	GO:0031977	12
Endoplasmic reticulum part	CC	GO:0044432	11
Golgi apparatus	CC	GO:0005794	9
Endoplasmic reticulum membrane	CC	GO:0005789	9
Photosystem I reaction center	CC	GO:0009538	5
NAD(P)H dehydrogenase complex (plastoquinone)	CC	GO:0010598	3
Catalytic activity	MF	GO:0003824	278
Oxidoreductase activity	MF	GO:0016491	96
Metal ion binding	MF	GO:0046872	89
Cation binding	MF	GO:0043169	89

Tetrapyrrole binding	MF	GO:0046906	30
Heme binding	MF	GO:0020037	25
Lyase activity	MF	GO:0016829	22
Hydrolase activity, acting on glycosyl bonds	MF	GO:0016798	19
Calcium ion binding	MF	GO:0005509	18
Hydrolase activity, hydrolyzing O-glycosyl compounds	MF	GO:0004553	17
Antioxidant activity	MF	GO:0016209	17
Monooxygenase activity	MF	GO:0004497	16
Peroxidase activity	MF	GO:0004601	14
Oxidoreductase activity, acting on peroxide as acceptor	MF	GO:0016684	14
Iron ion binding	MF	GO:0005506	14
Oxidoreductase activity, acting on paired donors, with incorporation or reduction of molecular oxygen	MF	GO:0016705	12
Oxidoreductase activity, acting on NAD(P)H	MF	GO:0016651	11
Oxidoreductase activity, acting on a sulfur group of donors	MF	GO:0016667	11
Enzyme inhibitor activity	MF	GO:0004857	10
protein disulfide oxidoreductase activity	MF	GO:0015035	8
Disulfide oxidoreductase activity	MF	GO:0015036	8
Carboxy-lyase activity	MF	GO:0016831	8
Transferase activity, transferring alkyl or aryl (other than methyl) groups	MF	GO:0016765	7
Peptidase regulator activity	MF	GO:0061134	7
Peptidase inhibitor activity	MF	GO:0030414	7
Oxidoreductase activity, acting on NAD(P)H, quinone or similar compound as acceptor	MF	GO:0016655	7
Endopeptidase regulator activity	MF	GO:0061135	7
Endopeptidase inhibitor activity	MF	GO:0004866	7
Oxidoreductase activity, acting on paired donors, with incorporation or reduction of molecular oxygen, NAD(P)H as one donor, and incorporation of one atom of oxygen	MF	GO:0016709	5
Glucosidase activity	MF	GO:0015926	5

Ribulose-bisphosphate carboxylase activity	MF	GO:0016984	4
Quinone binding	MF	GO:0048038	4
Phenylalanine ammonia-lyase activity	MF	GO:0045548	4
Oxidoreductase activity, acting on a sulfur group of donors, disulfide as acceptor	MF	GO:0016671	4
Beta-glucosidase activity	MF	GO:0008422	4
Ammonia-lyase activity	MF	GO:0016841	4
Acid phosphatase activity	MF	GO:0003993	4
Glucan endo-1,3-beta-D-glucosidase activity	MF	GO:0042973	3
Electron transporter, transferring electrons within the cyclic electron transport pathway of photosynthesis activity	MF	GO:0045156	3
Chitin binding	MF	GO:0008061	3
Carbon-oxygen lyase activity, acting on phosphates	MF	GO:0016838	3
Tyramine N-feruloyltransferase activity	MF	GO:0050366	2
Trans-cinnamate 4-monooxygenase activity	MF	GO:0016710	2
Terpene synthase activity	MF	GO:0010333	2
Oxidoreductase activity, acting on the CH-NH2 group of donors, NAD or NADP as acceptor	MF	GO:0016639	2
Hydroxymethylglutaryl-CoA synthase activity	MF	GO:0004421	2
Glutamate-ammonia ligase activity	MF	GO:0004356	2
Ammonia ligase activity	MF	GO:0016211	2
Acid-ammonia (or amide) ligase activity	MF	GO:0016880	2
3-hydroxyacyl-CoA dehydrogenase activity	MF	GO:0003857	2
3-deoxy-7-phosphoheptulonate synthase activity	MF	GO:0003849	2

BP: Biological Process; CC: Cellular Component; MF: Molecular Function
