# Supplementary material for: iTRAQ-Based Proteomics Analysis of Response to Solanum tuberosum Leaves Treated with the Plant Phytotoxin Thaxtomin A
Source: Int J Mol Sci. 2021 Nov 7;22(21):12036. doi: 10.3390/ijms222112036 (PMC8585116; doi:10.3390/ijms222112036)
Supplement: Supplementary file 1 [file ijms-22-12036-s001.zip › ijms-1405429-supplementary/Table S2. KEGG enrichment analysis of DEPs.pdf]

Table S2. KEGG enrichment analysis of DEPs

KEGG Description	Pathway ID	Protein Number
Protein processing in endoplasmic reticulum	sot04141	33
Phenylpropanoid biosynthesis	sot00940	26
Carbon metabolism	sot01200	26
Biosynthesis of amino acids	sot01230	24
Glutathione metabolism	sot00480	18
Photosynthesis	sot00195	17
Carbon fixation in photosynthetic organisms	sot00710	12
Phenylalanine, tyrosine, and tryptophan biosynthesis	sot00400	11
Plant-pathogen interaction	sot04626	11
Amino sugar and nucleotide sugar metabolism	sot00520	11
alpha-Linolenic acid metabolism	sot00592	10
Phenylalanine metabolism	sot00360	10
Fatty acid degradation	sot00071	10
Glyoxylate and dicarboxylate metabolism	sot00630	10
Glycolysis/Gluconeogenesis	sot00010	10
Alanine, aspartate, and glutamate metabolism	sot00250	9
Fatty acid metabolism	sot01212	9
Valine, leucine, and isoleucine degradation	sot00280	8
Peroxisome	sot04146	8
Starch and sucrose metabolism	sot00500	8
Ascorbate and aldarate metabolism	sot00053	7
MAPK signaling pathway - plant	sot04016	7
Phagosome	sot04145	7
RNA transport	sot03013	7
Spliceosome	sot03040	7
Butanoate metabolism	sot00650	6
Ubiquinone and other terpenoid-quinone biosynthesis	sot00130	6
Pentose phosphate pathway	sot00030	6
Pyrimidine metabolism	sot00240	6

Pyruvate metabolism	sot00620	6
Oxidative phosphorylation	sot00190	6
Ribosome	sot03010	6
Stilbenoid, diarylheptanoid, and gingerol biosynthesis	sot00945	5
Flavonoid biosynthesis	sot00941	5
Biosynthesis of unsaturated fatty acids	sot01040	5
Nitrogen metabolism	sot00910	5
Arginine biosynthesis	sot00220	5
Tyrosine metabolism	sot00350	5
Cyanoamino acid metabolism	sot00460	5
Protein export	sot03060	5
2-Oxocarboxylic acid metabolism	sot01210	5
Fructose and mannose metabolism	sot00051	5
Citrate cycle (TCA cycle)	sot00020	5
Cysteine and methionine metabolism	sot00270	5
Steroid biosynthesis	sot00100	4
Isoquinoline alkaloid biosynthesis	sot00950	4
Lysine degradation	sot00310	4
beta-Alanine metabolism	sot00410	4
Terpenoid backbone biosynthesis	sot00900	4
Endocytosis	sot04144	4
Synthesis and degradation of ketone bodies	sot00072	3
Tropane, piperidine, and pyridine alkaloid biosynthesis	sot00960	3
Arginine and proline metabolism	sot00330	3
Propanoate metabolism	sot00640	3
Galactose metabolism	sot00052	3
Glycerolipid metabolism	sot00561	3
Plant hormone signal transduction	sot04075	3
Glycerophospholipid metabolism	sot00564	3
RNA degradation	sot03018	3
Diterpenoid biosynthesis	sot00904	2

Taurine and hypotaurine metabolism	sot00430	2
Linoleic acid metabolism	sot00591	2
Other glycan degradation	sot00511	2
Sphingolipid metabolism	sot00600	2
Selenocompound metabolism	sot00450	2
Carotenoid biosynthesis	sot00906	2
Pentose and glucuronate interconversions	sot00040	2
Tryptophan metabolism	sot00380	2
Phosphatidylinositol signaling system	sot04070	2
Inositol phosphate metabolism	sot00562	2
Porphyrin and chlorophyll metabolism	sot00860	2
Fatty acid biosynthesis	sot00061	2
Ribosome biogenesis in eukaryotes	sot03008	2
Photosynthesis - antenna proteins	sot00196	2
Ubiquitin-mediated proteolysis	sot04120	2
Purine metabolism	sot00230	2
Brassinosteroid biosynthesis	sot00905	1
Sesquiterpenoid and triterpenoid biosynthesis	sot00909	1
Glycosylphosphatidylinositol (GPI)-anchor biosynthesis	sot00563	1
Indole alkaloid biosynthesis	sot00901	1
Glycosphingolipid biosynthesis - globo and isoglobo series	sot00603	1
Zeatin biosynthesis	sot00908	1
Glycosaminoglycan degradation	sot00531	1
AGE-RAGE signaling pathway in diabetic complications	sot04933	1
Sulfur relay system	sot04122	1
Arachidonic acid metabolism	sot00590	1
Mismatch repair	sot03430	1
Autophagy - other	sot04136	1
Biotin metabolism	sot00780	1
Thiamine metabolism	sot00730	1
Valine, leucine, and isoleucine biosynthesis	sot00290	1

Nicotinate and nicotinamide metabolism	sot00760	1
Ether lipid metabolism	sot00565	1
Homologous recombination	sot03440	1
DNA replication	sot03030	1
Nucleotide excision repair	sot03420	1
Sulfur metabolism	sot00920	1
Histidine metabolism	sot00340	1
Aminoacyl-tRNA biosynthesis	sot00970	1
Proteasome	sot03050	1
mRNA surveillance pathway	sot03015	1
