# Supplementary material for: iTRAQ-Based Proteomics Analysis of Response to Solanum tuberosum Leaves Treated with the Plant Phytotoxin Thaxtomin A
Source: Int J Mol Sci. 2021 Nov 7;22(21):12036. doi: 10.3390/ijms222112036 (PMC8585116; doi:10.3390/ijms222112036)
Supplement: Supplementary file 1 [file ijms-22-12036-s001.zip › ijms-1405429-supplementary/Table S3. Primers used in qRT-PCR.pdf]

Table S3. Primers used in qRT-PCR

Accession No.	Description	Sequence (5' to 3')	Fold Change
PGSC0003DMP400021162	Peroxidase	F: CCTCGTTGCTCTCTCTGGTG	4.103267
		R: CCACTACGCGGACAATTTGC	
PGSC0003DMP400059654	Peroxidase	F: AGTTGTCCAAGAACAGGAGGTG	3.459259
		R: GTGCAAAAGCCCTTTCTTTGC	
PGSC0003DMP400000820	Copper/zinc superoxide dismutase	F: ATGAAGTAAATGCAGCAACAGC	1.32017
		R: TTATTGGTAACCCGAAGAGGAG	
PGSC0003DMP400003634	Cysteine-rich secretory protein family	F: CCTAAAGCAAAATGGGGTTG	1.990189
		R: CACGGGCATCGTTGTGAA	
PGSC0003DMP400051894	Hsp90 protein	F: TATTATGAAAGCCCTTAACCCC	2.005472
		R: TGAGCCAATGAATTACTACCCTAC	
PGSC0003DMP400002803	Pathogenesis-related protein Bet vI family	F: TGCCCCTTCTAGGTTGTTCA	3.064176
		R: TCTCCCTCAGCCTCAATGTTC	
PGSC0003DMP400002693	Pathogenesis-related protein Bet vI family	F: CTGCTGGAGATGGAGGTTGT	2.554622
		R: ACAGAAGGATTAGCAAGGAGGTA	
PGSC0003DMP400062364	Glycosyl hydrolases family 17	F: GTCTGCTGGTGCATTTGGTG	2.116653
		R: TAGGTCCAGGCTTTCTCGGA	

PGSC0003DMP400053803	NAD	F: GCACTATGATTTCCCCGACTT<br>R: TTCCATTCCTCTACTTGCTCCT	1.463713
PGSC0003DMP400034650	ATPase family associated with various cellular activities	F: GCACAACAGCAGCTTATCGG<br>R: TGCCCTTCTTCAGCACTCC	0.9
PGSC0003DMP400003405	Phosphoglucomutase	F: ATTGAGCACAAAATACCGACTG<br>R: CATGCCAACTTCCCTCCTACA	1.010274
PGSC0003DMP400025999	Acetyltransferase	F: TGCTCCTCAACTTCCTACACCA<br>R: TCGTAGCCAGACGGACTCTT	7.174355
PGSC0003DMP400036207	Ras family	F: TCCTCCAACATTTGCCCATT<br>R: CCACTCTATTCCCCATCACTTT	1.309073
PGSC0003DMP400046968	Mitochondrial carrier protein	F: CCCTTGGTAGGAGCCAAATAT<br>R: CGTTGCTGTAATGGTGGAGAA	1.762727
PGSC0003DMP400022299	Peroxidase	F: TGACAGGGACTAACGGTGAAA<br>R: TGCGCTAACTGAACGAAACTAA	3.266057
PGSC0003DMP400000965	Carbonic anhydrase	F: TTTGTGGTGGGCTTTGGTAG<br>R: TCTGGGAATCCTCTTGTTGCT	0.773168
PGSC0003DMP400026922	Short chain dehydrogenase	F: AAAGAGCCTTCGTTCACAGC<br>R: TGGCCTTTGGAGGTACATTT	0.694582

PGSC0003DMP400041818	Ribulose-1,5-bisphosphate carboxylase small subunit	F: TCATTGCCTACAAGCCAGAAG<br>R: CGGAATCGAGGAAAATACACC	0.714299
